# Improved Ocular Bioavailability of Moxifloxacin HCl using PLGA Nanoparticles: Fabrication, Characterization, *In-vitro* and *In-vivo *Evaluation

**DOI:** 10.22037/ijpr.2021.114478.15054

**Published:** 2021

**Authors:** Fahim Ullah Khan, Fazli Nasir, Zafar Iqbal, Steven Neau, Ismail Khan, Mohammad Hassan, Muhammad Iqbal, Aman Ullah, Sumaira Irum Khan, Mirina Sakhi

**Affiliations:** a *Department of Pharmacy, University of Peshawar, Peshawar-25120, Pakistan. *; b *Department of Pharmacy, City University of Science and Information Technology Peshawar, Peshawar- 25000, Pakistan. *; c *Department of Pharmaceutical Sciences, Philadelphia College of Pharmacy and Science, Philadelphia-PA 19104, USA. *; d *Department of Pharmacy, University of Swabi, Swabi- 23561, Pakistan.*; e *Department of Statistics, University of Peshawar, Peshawar-25120, Pakistan. *; f *Department of Pharmacy, Abasyn University Peshawar, Peshawar- 25000, Pakistan.*

**Keywords:** Polymeric nanoparticles, Moxifloxacin hydrochloride, Ocular bioavailability, Pharmacokinetics, Irritation

## Abstract

Improving the bioavailability of a drug at the ocular surface presents a profound challenge. Due to ocular physiological barriers, conventional eye drops exhibit poor bioavailability of drugs. Sustained-release nanoparticles may improve the residence time and hence increase absorption of the drug from the corneal surface. The current study focuses on the development of a nanoparticle-based system for the ophthalmic sustained delivery of moxifloxacin, to enhance ocular retention and bioavailability of the drug. PLGA was used as the matrix-forming polymer in the nanoparticle formulation. Nanoparticles were manufactured using a double emulsion (w/o/w) solvent evaporation technique. The formulation was optimized based on physicochemical properties, including size, polydispersity index, and stability. Nanoparticles were also evaluated for *in-vitro* drug release and pharmacokinetic evaluation in a rabbit model. The optimized formulation exhibited a relatively high initial release rate for six hours followed by sustained release of a drug via diffusion. The *in-vivo* ocular tolerance studies confirmed that moxifloxacin-loaded PLGA nanoparticles were non-irritating to the eye. The pharmacokinetic studies revealed that the nanoparticles provided a high C_max_, AUC, MRT, and low clearance rate when compared to commercial eye drops. It can be concluded that such PLGA nanoparticles offer the potential for improved bioavailability of moxifloxacin HCl.

## Introduction

Topical application of drugs is usually preferred to treat ocular diseases because it provides rapid and localized action with minimum systemic toxicity (1, 2). However, the success rate of the topical route is limited due to various factors such as rapid tear turnover diluting the inserted product, blinking and squeezing the eyelids causing drainage down the cheek, a short drug residence time in the cul-de-sac due to nasolacrimal drainage which is encouraged by squeezing the eyelids, and low drug permeability due to the corneal membrane barrier (3-5). In most cases, the corneal route was the major pathway for drug absorption (6, 7). As a result of these challenges, less than 5% of drug administered topically to the surface of the eye is expected to reach intraocular tissues (1, 3, 5, 7 and 8). To achieve therapeutic drug levels, frequently repeated drug instillation is necessary, which may lead to drainage via the nasolacrimal duct to the sinuses and absorption at the mucus membrane; the result is systemic toxicity and poor patient compliance (2, 3 and 5). A major challenge in topical ocular therapy, therefore, is to maintain an optimum drug concentration for a long time at the ocular surface (5).

Polymeric nanoparticles have been investigated for ocular drug delivery to improve ocular bioavailability (9-12). Nanoparticles have a sub-micron size that is easily tolerated by patients and can reside in the cul-de-sac for a long time if mucoadhesive (7, 13). Polymeric nanoparticles can control drug release, prolong residence time in the cul-de-sac by bioadhesion, and prevent drug washout due to tear dynamics to ensure optimal contact of the formulation with the ocular mucosa to enhance drug delivery and thus improve bioavailability at the ocular tissues (14, 15). Poly(lactic-co-glycolic acid) (PLGA) is a biodegradable and biocompatible polymer that is extensively used for nanoparticle-based controlled drug delivery and site-specific drug targeting (12, 16 and 17). A sparfloxacin-containing PLGA ophthalmic nanosuspension demonstrated improved precorneal retention time and ocular permeation (18). To treat an ocular infection, drug penetration at the target site, potency and drug, product safety are the three essential aspects of therapeutic control (19). Moxifloxacin, a fourth-generation fluoroquinolone antibiotic, is available as a 0.5% w/v ophthalmic solution that possess antibacterial potency and higher ocular tissues penetration (20). The systemic and ocular safety profile of moxifloxacin exhibits the recognized low risk of quinolone-related toxicity. Moxifloxacin has similar cytotoxicity potential as that of the other drugs from this family in *in-vitro* studies using human or rabbit corneal epithelial cells or keratocytes (19). Moxifloxacin HCl (MX) is widely used in the treatment of bacterial conjunctivitis, keratitis, kerato conjunctivitis and prophylactically in refractive and cataract surgeries (21-24). The 0.5%w/v MX eye drops provide effective dosing in 01 drop three times a day for seven days to treat acute bacterial conjunctivitis. In addition, delivery of moxifloxacin to the ocular surface has been investigated using contact lenses (3), nanoparticles (10), drug matrix inserts prepared using bioadhesive polymers (25), a gel-forming ophthalmic solution (26-28), and a nanosuspension (29). In general, these approaches will blur or obscure vision, but nanoparticles, including the nanosuspension, should settle in the cul-de-sac and not adversely affect vision. For ocular devices intended for sustained drug delivery to the ocular surface, nanoparticles are better tolerated, although larger microparticles demonstrated slower elimination from the cul-de-sac (30). An amphotericin-loaded Eudragit^®^ RS-100 nanosuspension with a particle size range of 150-290  nm proved to be an efficient drug delivery vehicle for 24  h with no ocular irritation observed after topical instillation into the rabbit eye (31). Even over a four-week study, nanosuspension formulations were well tolerated (32).

The goal of the present work was to formulate MX-loaded PLGA nanoparticles capable of sustaining drug release at the ocular surface to enhance MX bioavailability. Physicochemical characterization, *in-vitro *drug release, stability study, and *in-vivo* bioavailability to aqueous humor were evaluated. Comparisons were made with the performance of a commercially available ophthalmic solution as appropriate.

## Experimental

*Materials*

Moxifloxacin hydrochloride of 99.9% purity was obtained from Dr. Raza Pharma, Pvt., Ltd., Peshawar, Pakistan. Poly(lactide-co-glycolide) with a 75:25 ratio (PLGA Resomer^®^ RG 752H) was purchased from Evonik (Essen, Germany). Other excipients and reagents, including poly(vinyl alcohol) (PVA, Sigma-Aldrich, St. Louis, MO, USA), Tween80 (Daejung, Busan, Korea), dichloromethane (DCM, Scharlab Chemie, Sentmenat, Spain), mannitol, sucrose, and glucose (Merck, Darmstadt, Germany) were used as received.

*Methods*

*Drug Excipients Compatibility*

The FTIR spectra of MX, PLGA and their 1:1 physical mixture were obtained to determine any possible interaction (10). Samples were prepared as a KBr disc. Using a Perkin Elmer spectrum BX FTIR (PerkinElmer, Waltham, MA, USA), the percent transmittance (%T) was recorded in the spectral range 400-4000 cm^-1^.

*Preparation of Nanoparticles*

Nanoparticles were formulated using a modified double emulsion-solvent evaporation technique (33, 34). Briefly, 2 mL of aqueous 0.25% w/v drug solution was poured into 4 mL of PLGA solution with its concentration in dichloromethane varied over 0.0625-1.25% w/v. This mixture was sonicated for 1 min at 100% amplitude using a probe sonicator (Soniprep 150 ultrasonic disintegrator, MSE (UK) Ltd, London, UK) to obtain the primary w/o emulsion. The primary emulsion was added to different phosphate buffer solutions (10 mL) containing different concentrations of PVA, and sonicated again for 2 min at 100% amplitude to obtain a w/o/w emulsion. After sonication, the emulsion was stirred until all the DCM was evaporated. Drug-loaded nanoparticles were collected by centrifugation at 16, 000 rpm at 4 ^o^C for 20 min. The nanoparticles were washed three times with distilled water and then lyophilized using a Cryodos-50 Freeze Dryer **(**Telstar North America, Bristol, PA, USA), adding 5% (w/v) mannitol as a lyoprotectant.

Particle size and Polydispersity Index (PDI)

The particle size and the PDI of the nanoparticles were determined by dynamic light scattering using a Zetasizer, Nano ZS-90 (Malvern Panalytical Ltd, Malvern, UK). The nanoparticle samples were diluted in distilled water and dispersed by vortex for analysis at room temperature with a 90° scattering angle. Each of the measurements was performed in triplicate and the mean and standard deviation were calculated.

Zeta Potential

The zeta potential of the nanoparticles was determined by **laser Doppler micro-electrophoresis **using a Zetasizer, Nano ZS-90 (Malvern Panalytical Ltd, Malvern, UK.). Each of the measurements was carried out in triplicate and the mean and standard deviation were calculated. 

*Stability Studies of the Formulations*

The stability of the nanoparticles in terms of size and polydispersity was evaluated by storing the nanoparticles at different temperatures (27-30 °C and 4-8 °C) for 8 weeks. Samples were withdrawn at regular time intervals from these nanosuspensions to be assessed for any change in size and polydispersity.

X-Ray Diffraction 

To assess the amorphous or crystalline nature of the pure drug, PVA, PLGA, and drug-loaded nanoparticles, the XRD pattern was measured using a JDX-3532X-ray diffractometer (JEOL Ltd., Tokyo, Japan). Samples were investigated over a 2θ angular range of 10-40 degrees.

Scanning Electron Microscopy

Drug-loaded nanoparticles were analyzed for shape and surface morphology with a JSM-5910 scanning electron microscope (JEOL Ltd., Tokyo, Japan). Each sample was prepared by spreading a nanoparticle suspension on adhesive carbon tape glued to a stub. A gold layer was applied under a vacuum to the nanoparticle surfaces using an SPI-Module Sputter Coater (SPI Supplies, West Chester, PA, USA) for 90 s. The samples were then observed at various magnifications and images were captured.

Drug Loading and Release

Entrapment efficiency

The percent entrapment efficiency of moxifloxacin Hcl in nanoparticles was determined by centrifuging a 2 mL aliquot of freshly prepared nanoparticles at 16,000 rpm at 4 °C for 20 min (Centurion Scientific, Chichester, UK). The supernatant containing the unincorporated drug was measured using UV spectrophotometry at 299 nm. The entrapment efficiency (%) was calculated using Equation 1:

$\mathrm{Entrapment Efficiency}\left(\%\right)=\frac{\mathrm{Total drug mass}-\mathrm{Drug mass found in the supernatant}}{\mathrm{Total drug mass}}\times 100\%$ Equation 1. 

*In-vitro Drug release*

The *in-vitro* release of drug from MX-PLGA nanoparticles was observed in simulated tear fluid (STF); sodium chloride (0.67 g), sodium bicarbonate (0.2 g) and calcium chloride dehydrate (0.008 g) in distilled water qs to make 100ml (pH 7.4). Briefly, a sample of lyophilized nanoparticles equivalent to 1 mg of the drug, was dispersed in 2 mL STF and filled into a dialysis membrane bag with a molecular weight cut off of 12-14 kDa. The dialysis bag was then placed into 100 mL of STF in a water bath shaken at 50 ± 1 rpm and temperature maintained at 37 ± 0.5 °C. A 2 mL sample was withdrawn from outside the dialysis bag at regular time intervals. Each sample was replaced with the same volume of release medium that had been held at the same temperature. The withdrawn samples were then analyzed for MX contents using UV spectrophotometry. Each of the measurements was performed in triplicate and the percent cumulative drug released was calculated using the following equation (13):

$\mathrm{Cumulative drug released}(\%)=\frac{D_{t}}{D_{T}}\times 100\%$

Equation 2.

where Cumulative drug released (%) is the cumulative percent of the total drug released at a particular time point, t, in the release study, D_t _is the cumulative mass of drug released at time t, and D_T_ is the total amount of drug in the MX-PLGA nanoparticle sample for analysis.

*Drug release mechanism*

To predict the mechanism of drug release from the nanoparticles, different mathematical models were fitted to *in-vitro* drug release data. The models employed in this study are:

Higuchi Model$\frac{M_{t}}{M_{\infty }}=k_{1}\sqrt[{2}]{t}+c$

Peppas and Sahlin MtM∞=k1t+k2t2+c

$\mathrm{Hixson}-\mathrm{Crowell}{\left(\frac{M_{t}}{M_{\infty }}\right)}^{\frac{1}{3}}=1-k_{1}t$

Weibull MtM∞=1-exp-t-tlagk1k2 – c

k_1_= shape of dissolution curve while k_2_= parameter that describes the time

*In-vivo Evaluation*

Animals

White adult New Zealand rabbits weighing 2.2 ± 0.2 kg and free from any sort of inflammation or any other abnormality were used in this study. Rabbits were acclimated to the standard laboratory conditions i.e. 61-72 °F and 55 ± 10% RH. Rabbits were fed with fresh vegetables and allowed free access to water. The study was reviewed and approved by the Committee for Ethics in Research, Department of Pharmacy, University of Peshawar [Approval No. 06/EC-17/Pharm].

Ocular tolerance test

The safety and biocompatibility of the nanoparticles were evaluated by conducting an ocular irritation test. Therefore 30µL of the optimized nanoparticles (MX 20), negative control (normal saline), or commercial eye drops were instilled topically into the conjunctival sac of one eye of the selected rabbits from each group. The second eye was used as a control. The ocular tissues were observed at certain times for conjunctival redness, discharge, and any evidence of inflammation on a clinical evaluation scale of 0-3, 0-4 and 0-3 respectively (35).

*Pharmacokinetic studies*

Animals were divided into four groups of 10 rabbits each. Lyophilized nanoparticles were dispersed in normal saline to make a 0.5% w/v MX suspension. Group 1 received 30µl of formulation MX 10, Group 2 received the same volume of MX 15, Group 3 received 30µl of MX 20, and Group 4 received 30µl of the commercial formulation. The drug was instilled into the conjunctival sac of both eyes and then 100 µL samples of the aqueous humor were collected from each group using a 27 gauge needle with a 1 mL syringe by sacrificing a corresponding rabbit at a certain time (36). The aqueous humor samples were stored at -20 °C until analyzed for drug content by HPLC-UV (37). 

Various pharmacokinetic parameters, including time to reach maximum concentration (T_max_), maximum drug concentration in the aqueous humour (C_max_), the area under curve (*AUC*_0-t_) and mean residence time (MRT) were calculated for these formulations using PK Solutions pharmacokinetic software from Summit PK in Montrose, CA, USA.

*Statistical Analysis*

The data obtained are presented as the mean ± SD with n = 3. Analysis of variance (ANOVA) was applied to the data using Microsoft Excel to access the level of significance of any differences. The difference was deemed significant if *p < *0.05. Regression analysis was conducted using SigmaPlot v. 12.5 (SysStat Software, Inc., San Jose, CA).

## Results and Discussion

Loading moxifloxacin hydrochloride into hydrophobic nanoparticles results in low encapsulation efficiency (38). To solve this problem, PLGA nanoparticles were prepared using various techniques and altering formulation parameters such as a drug to polymer ratio (D:P) and surfactant (PVA) concentration, and optimized formulations for ophthalmic delivery were developed.

*Drug Excipients Compatibility Studies*

**Figure 1:**
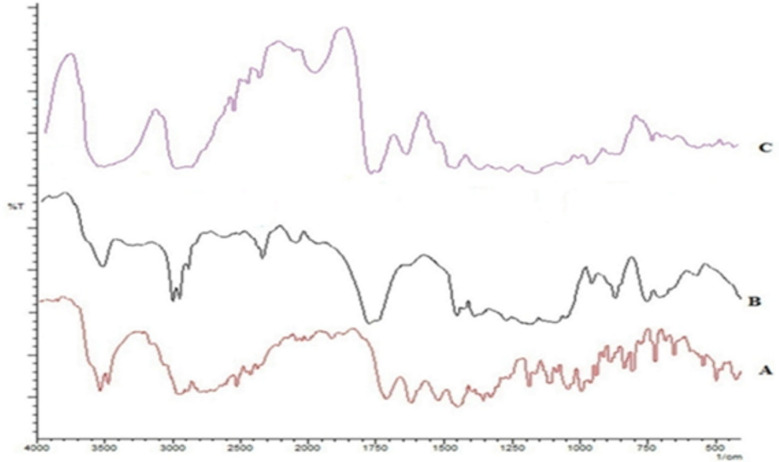
Overlay of FTIR spectra of (A) moxifloxacin hydrochloride, (B) PLGA, and (C) 1:1 physical mixture

An interaction between MX with PLGA was assessed through FTIR spectroscopy. The spectra are shown in Figure 1. The spectrum of MX gave characteristic peaks at 1706 cm^-1 ^due to C=O stretching in the carboxylic acid group, C-N stretching at 1350 cm^-1^, and 1620, 1520, and 1460 cm^-1^ owing to aromatic C=C stretching. PLGA characteristic bands appeared at 1765 cm^-1^ for carbonyl C=O stretching, at1180 cm^-1^ for C-O stretching and at 956 cm^-1^for OH bending. The presence of peaks for each of these characteristic groups in the spectrum for a 1:1 physical mixture authenticates the lack of any interaction or incompatibility of the drug with the excipients used.

*Particle Size, PDI, and Zeta Potential*

**Figure 2:**
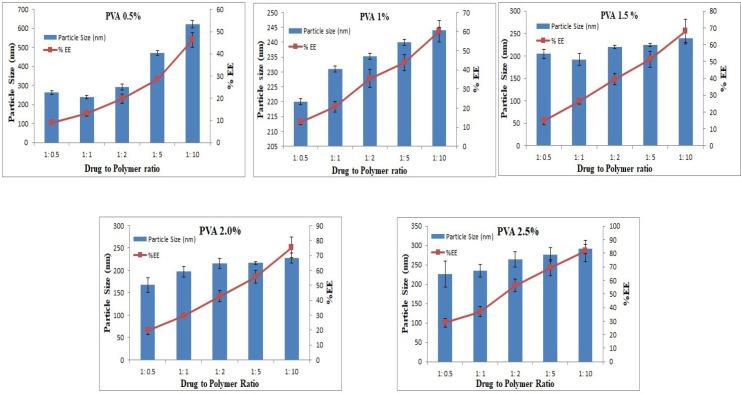
The effects of the drug to PLGA ratio on mean Particle Size and percent entrapment efficiency

Small-sized particles are usually preferred in ophthalmic delivery, since larger particles cause discomfort and irritation. The mean sizes of the prepared formulations ranged from 167.4 - 622.4 nm. The particle size increased as D:P was increased from 1:1 to 1:10. Increasing the polymer concentration increases the viscosity of the external phase of the primary emulsion which results in larger primary emulsion droplets in the w/o/w emulsion and resistance to size reduction to the nanoparticle level (39). Larger particles were produced with 0.5% w/v PVA, and the size decreased with an increase in the PVA concentration. For instance, 220, 204, and 167 nm particles were produced with 1, 1.5, and 2% w/v PVA, respectively. However, at 2.5% PVA concentration, the particle size increased to 227 nm, as shown in Figure 2. The results presented here are in accordance with previously reported results, although only two levels for the polymer and PVA were studied (40).

The polydispersity index (PDI) was low for each of the formulations, over the range of 0.09-0.48. PDI is a measure of homogeneity and uniformity of the particle size and particles ranging between 0.15–0.3 are considered homogeneous (41).

The zeta potential results revealed that each type of nanoparticle carried a negative charge, ranging from -1.3 to -14.54 mV. The negative charge on PLGA nanoparticles is due to the presence of carboxylic end groups on the ends of each PLGA backbone (39).

*Stability Studies*

**Figure 3:**
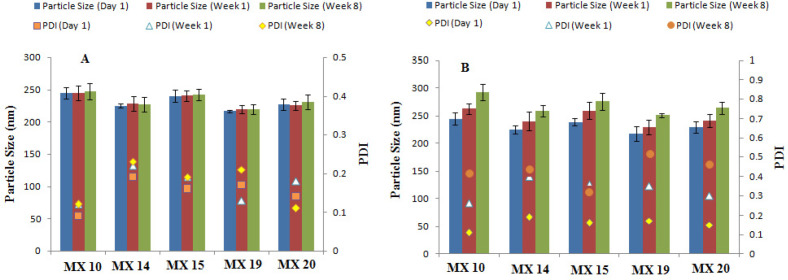
Stability of nanoparticle formulations after storing at (A) 4-8 °C and (B)27-30°C

Stability studies of the freeze-dried nanoparticles stored at refrigerator temperature (4-8 °C) revealed no significant changes in particle size or PDI when stored for 2 months (Figure 3) as low temperatures decrease the kinetic energy and thus inhibit particle aggregation (42). Upon storage at room temperature, a significant change in particle size and PDI were detected, thus suggesting storage of nanosuspensions at 4-8°C to prevent any changes in the nanoparticle size and PDI.

*X-Ray Diffractometry*

**Figure 4:**
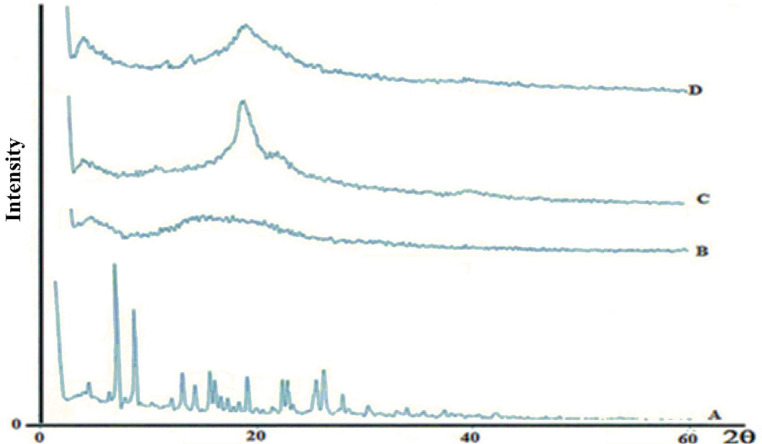
X-ray diffractograms of [A] MX, [B] PLGA, [C] PVA, [D] MX-loaded nanoparticles

XRD spectra of the freeze-dried nanoparticles showed no intensity peaks for the drug, although the broad peaks associated with PLGA and PVA are evident, suggesting the lack of crystalline drug in the polymeric nanoparticles or that the drug is molecularly dispersed in the polymer matrix (Figure 4). Although amorphous polymers show only broad halos in diffractograms (43), the multiple broad peaks in Figure 4, confirms that PLGA is a semi-crystalline polymer (44). The height of the PLGA peak is diminished in Figure 4D because the level of PLGA in the mixture is less than in the pure PLGA.

*Scanning Electron Microscopy*

**Figure 5:**
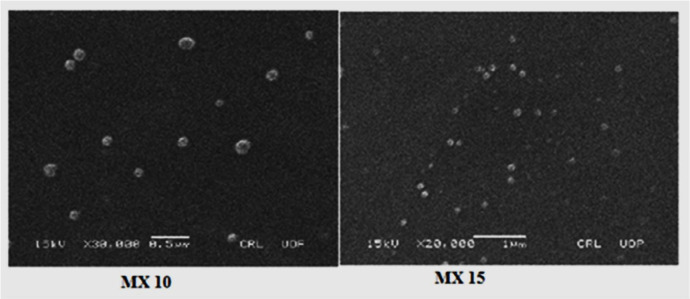
SEM images of MX-loaded nanoparticles (MX 10, MX 15)

The morphology of the nanoparticles is shown in SEM images (Figure 5) which reveal that the nanoparticles are spherical with smooth surfaces. This is in agreement with reports on the spherical appearance of PLGA nanoparticles produced by a similar technique (45).

*Drug Loading and Release*

The percent encapsulation efficiency, EE (%), of nanoparticles was significantly affected by the polymer concentration as it is the most influential parameter for this response. Results show that the highest EE (%) of 81.7% was observed at a D:P ratio of 1:10 and 2.5% PVA concentration. When D:P was 1:1, the EE (%) was 13.2%, whereas with increasing the D:P ratio, higher EE (%) was achieved, as shown in Figure 2.

Based on the particle size, polydispersity and EE (%) values, five formulations with the smallest particle size and highest encapsulation efficiency, namely MX 10, MX 14, MX 15, MX 19, and MX 20, were selected for further *in-vitro* and *in-vivo* evaluation.

**Figure 6:**
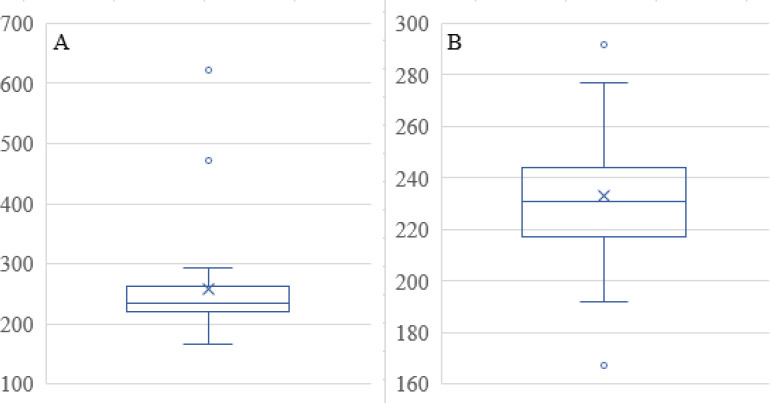
Box and Whisker Plots for Mean Size Data.(A) Full set of data, (B) Data without the outliers evident in A that established new ranges. The X presents the mean value and the top and bottom whiskers on the vertical line show the range outside of which outlier(s) exist. The horizontal line in the box indicates the median value for the mean size data

The Mean Size in Table 1 has three outliers that were removed. The two data points that were removed from the data set are evident in the Box and Whisker Plot in Figure 6A, namely 471 and 622, as data points that exceed the upper whisker in the plot. The remaining data reveal two more values for outliers in Figure 6B. Since there are two examples of the value that is too high in Figure 6B, at 292 nm, neither of these was removed, even though the low value, specifically 167, was considered an outlier and removed from the data set before further data analysis. Predicted Mean Size as a function of the factor levels with the squared response data transforms provided an excellent fit to the data. Equation 3 included two-factor interaction and quadratic terms:

(Mean Size)^2^ = 85000 – 2470A – 82400B – 82400C – 17000AB – 750BC + 171AC + 27.8A^2^ + 26800B^2^ + 25200C^2^

Equation 3.

**Figure 7:**
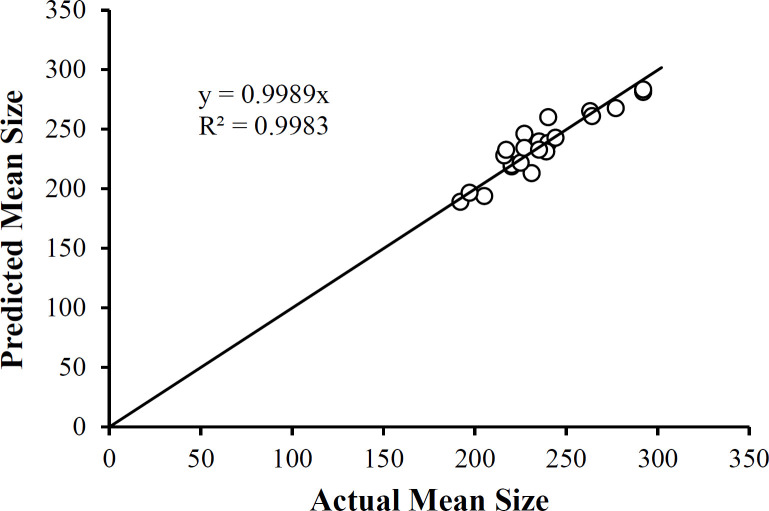
A Plot of Mean Size Data Predicted Using Equation 1 as a Function of the Actual Mean Sizes

Where A = PLGA (mg), B = Drug to Polymer Ratio, C = PVA (% w/v) using the actual factor values. Equation 3 reveals that main factors B and C profoundly influence the Mean Size not only individually but at the quadratic terms for these two factors. In addition, the two-factor interaction terms in A and B is also substantially influencing the Mean Size. Therefore, each of the three studied factors affects the mean size of the nanoparticles. A plot of the predicted mean size values as a function of the actual mean size values (Figure 7) shows the correlation between the two sets of data. Not only is the correlation coefficient high (R^2^ = 0.8576), but the slope of the linear regression equation reveals the identity relationship between the two sets of data (slope = 0.9989). Note that the data clusters around 250 nm, comparable to that reported by Dillen *et al. *(46).

**Figure 8:**
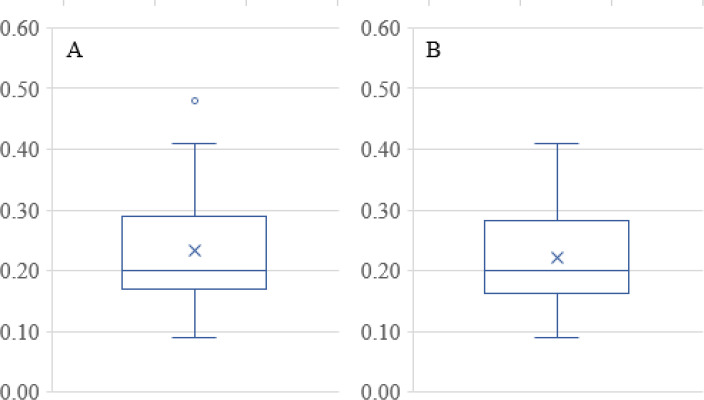
Box and Whisker Plots for PDI data. (A) Full data set revealing a single outlier at 0.48, (B) Data without 0.48 showing no further outliers

**Figure 9:**
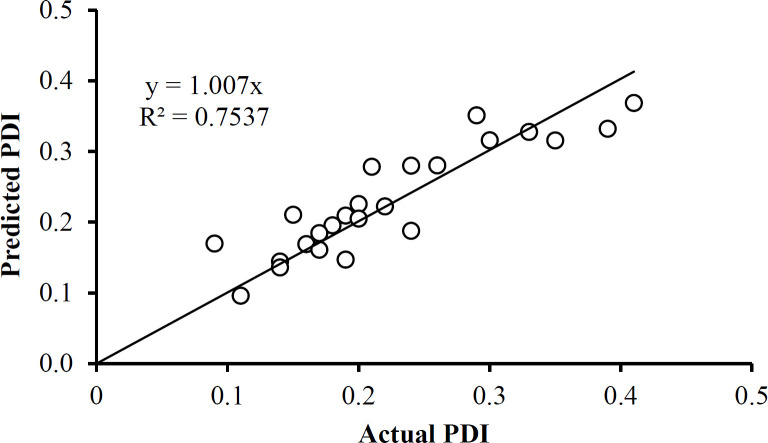
Predicted values for PDI as a function of the actual PDI values. The linear relationship is presented in Equation 4

The PDI data in Table 1 has only one outlier, at 0.48 in the Box and Whisker Plot in Figure 8A After eliminating this data point, it was found that no further outliers were evident (Figure 8B). The PDI data was described well without 0.48 by Equation 4. Note that the data fall essentially on the identity line in Figure 9. The equation indicates that the only way in which factor A affects PDI is by a two-factor interaction with factor B. Factors B and C influence this response as main factors, in two-factor interaction terms, and quadratic terms, revealing the importance of the proper selection of the drug-to-polymer ratio and the concentration of PVA in the external aqueous phase of the w/o/w emulsion.

PDI = 45.9 + 0.0145A + 0.523B + 0.105C – 9.22AB –0.0366BC + 0.0000161AC – 0.000190A^2^–0.170B^2^ – 0.0474C^2^

 Equation 4.

There were no outliers in the zeta potential or the entrapment efficiency data in Table 1. Each of these responses can be described by a corresponding multiple regression equation:

Zeta Potential = 104 – 1.05A – 38.2B + 10.2C – 18.2AB – 0.143BC – 0.0106AC + 0.0126A^2^ + 12.3B^2^ – 2.47C^2^

Equation 5.

Entrapment Efficiency (%) = 116.1 – 0.629A – 44.7B + 20.8C – 15.8AB – 4.79BC + 0.00550AC + 0.0146A^2^ + 14.0B^2^ – 0.902C^2^

Equation 6.

**Figure 10:**
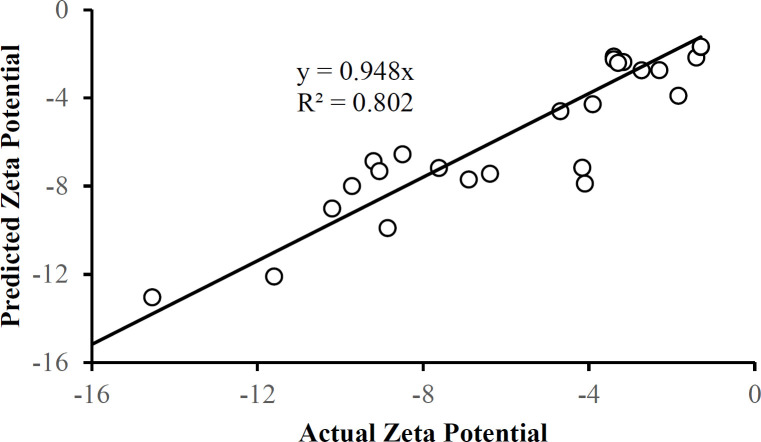
The plot of predicted values for the zeta potential based on Equation 5 as a function of the actual zeta potential values

**Figure 11:**
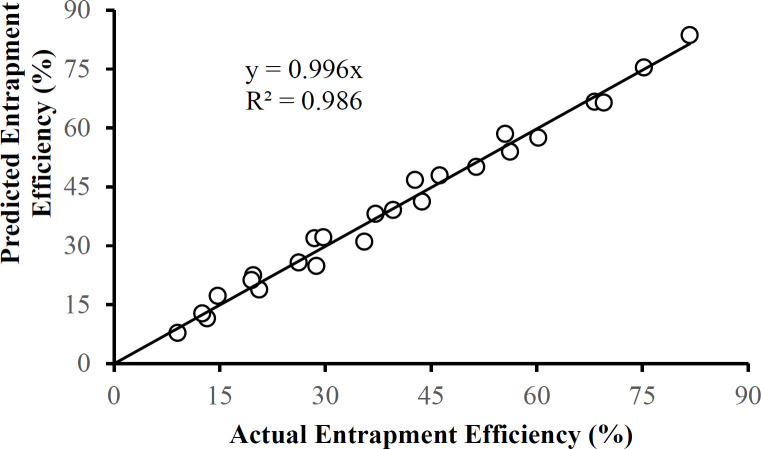
Entrapment efficiency predicted using Equation 6 as a function of the actual entrapment efficiency

Figures 10 and 11 show that the data is described well by the corresponding equations and that in each case the responses follow the identity line. The drug-to-polymer ratio (Factor B) has the most profound effect on zeta potential as a main factor term, in its quadratic term, and the AB two-factor interaction term. The level of PVA in the external aqueous phase (Factor C) also markedly influences this response in its main factor term. The drug-to-polymer ratio substantially affects the entrapment efficiency as the main factor term, in its quadratic term, and the AB two-factor interaction term. As the main factor term the PVA concentration (Factor C) also markedly affects the entrapment efficiency. In this way, each of the factors markedly affects the entrapment efficiency.

*In-vitro Drug Release*

**Figure 12:**
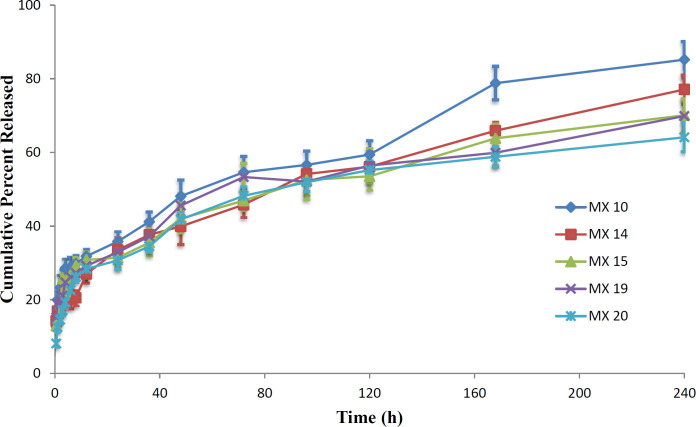
*In-vitro *Release profiles for MX-loaded nanoparticles

*In-vitro *drug release profile for the preferred MX-loaded nanoparticles is shown in Figure12. Each of the formulations released the drug in a biphasic manner; with a high release rate at first followed by sustained drug release. This is confirmed by when the mathematical models were fitted to the data, with results in accordance with previous reports (47, 48). The high release rate is observed up to 6h, followed by consistent release up to 240 h. The initial high drug release rate may be attributed to the surface drug (38, 49). The release efficiency of the products, RE_24h_, equal to the cumulative percent of drug released at 24 h, decreased when the drug-to-polymer ratio or the PVA concentration was increased. The RE_24H _obtained for the formulation MX10, MX14, MX15, MX19, and MX 20 was 35.8, 33.5, 31.4, 32.9, and 30.7%, respectively. The trend of a decrease in the drug released can be attributed to the higher polymer concentrations that increase the viscosity of the organic phase that result in less diffusion of the drug from the internal phase to the external phase (50). Therefore, less drug migrates to the surface of the particle during the preparation of the nanoparticles.

**Table 1:** Formulation parameters and physicochemical characteristics of various formulations.

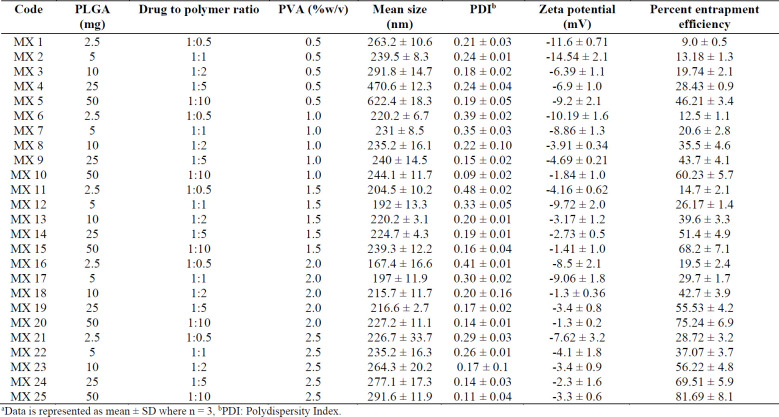

**Table 2:** Model fitting to the percent released data for MX formulations

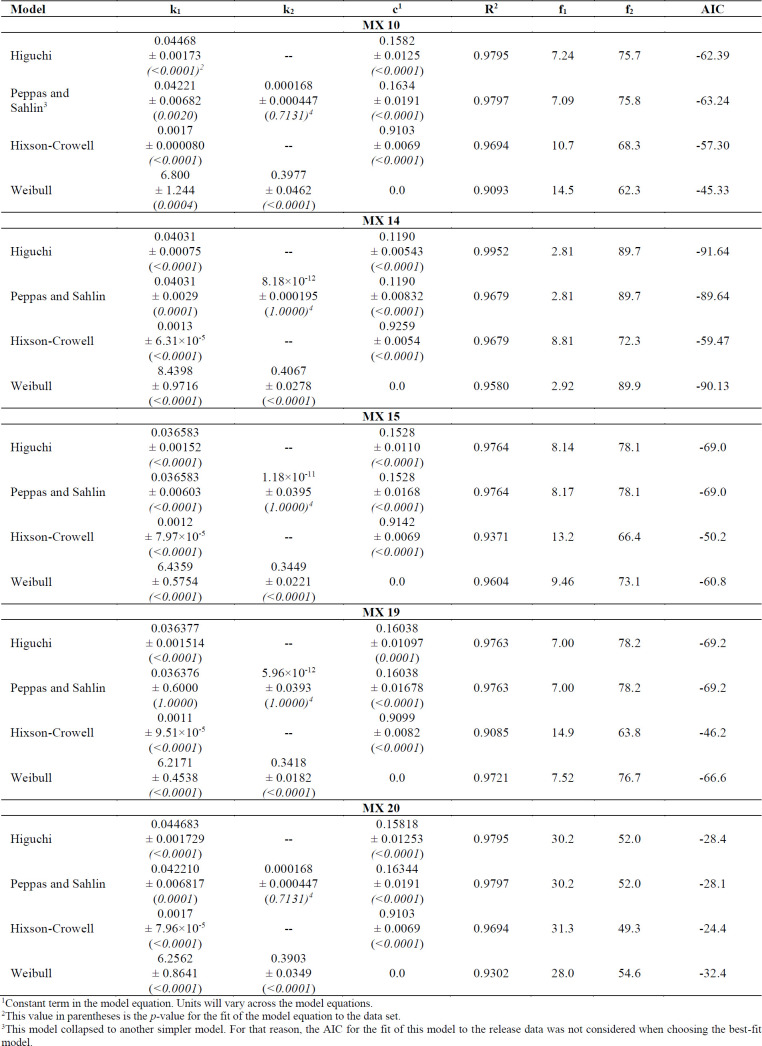

**Table 3:** Grading of macroscopic signs observed after the ocular tolerance studies for the MX 20 nanoformulation with comparison to commercial eye drops and normal saline control.

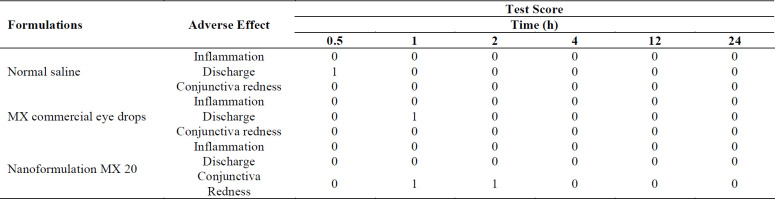

**Table 4:** Pharmacokinetic parameters for the commercial ophthalmic 0.5% w/v solution and nanoformulations of moxifloxacin hydrochloride (n = 3).

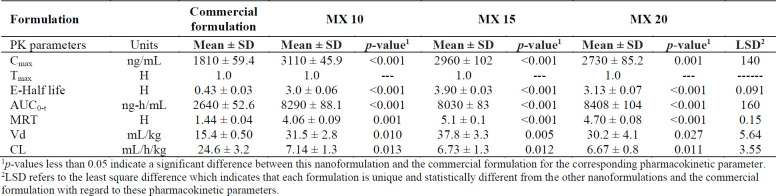

*Drug Release Kinetics*

Different release models were fitted to the MX nanoparticle release profiles to predict the release mechanism. Since there is a burst release of the drug up to about 6 h, it can be assumed that the release mechanism in effect during that first 6 h is different from the release mechanism that is in effect from 6-240 h in the profile. What is of importance is the mechanism when steady-state drug release is evident, which would exist in the 6-240 h range of the profile, and the value of the burst release is estimated by the model fitting. Therefore, the model equations were fitted only to the 6-240 h data of the release profile and the measure of the burst release is the value at 6 h predicted by the fitted model equation.

The Akaike Information Criterion (AIC) was used to predict the model that best fit the release data with the fewest estimated parameters. The data in Table 2 indicate that the Higuchi model provided the best fit to the release data as obvious from the more negative value for the AIC for formulations MX 10, MX 14, MX 15, and MX 19. The exception was data for MX 20 which is best described by the Weibull equation. This confirms that, even though an apparent biphasic release profile was evident, diffusion of the release medium into the drug-containing nanoparticles, dissolution of the drug, and diffusion of the dissolved drug out of the particles is still the expected release mechanism for most of the entrapped drug.

The value ‘c’ indicates the fraction of drug released due to the rapid release rate in the first 6 h. Based on the fit of the Higuchi model equation to the release data, approximately 16% of the drug was released during the initial burst of the drug from the nanoparticles. 

*In-vivo Studies*

*Ocular Tolerance Studies*

Administration of moxifloxacin 0.5% resulted in a significant decrease in pupil size compared with baseline (*p = *0.004), believed to be due to a greater release of endogenous prostaglandins (51). Toxicity studies in rabbits demonstrated a substantial margin of safety for topically administered 0.5-3.0% moxifloxacin solutions, low ocular irritation potential, and no evidence of ocular or systemic toxicity (52). The hen’s egg test on chorioallantoic membrane (HET-CAM) has been developed as a toxicological method to determine ocular irritation potential (53). Sparfloxacin-loaded PLGA nanoparticles of 180-190 nm particle size were tested using the HET-CAM assay and earned a 0 score over an 8 h period for the nanosuspension (18). Therefore it is not surprising that visual examination after the ocular irritation test revealed that no detrimental effects or irritation occurred with any of the formulations, indicating their ocular safety. A minimal redness of the conjunctiva was recorded with the nanoformulations that also resolved within 2 h. The results are presented in Table 3. The obtained results confirm that the nanoformulations can be used for ocular instillation because they were safe, biocompatible with the ocular tissues, and non-irritating.

*Pharmacokinetic Studies*

**Figure 13:**
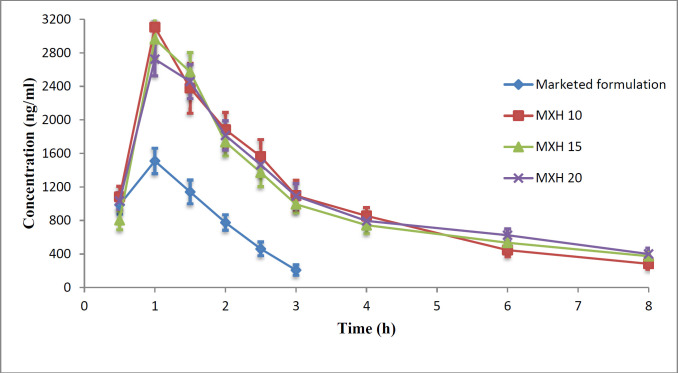
Aqueous humour concentration after MX-loaded nanoparticle or 0.5% w/v commercial eye drop instillation into a rabbit’s eye

The commercial product or a nanoformulation at 0.5% w/v MX content was instilled into each rabbit’s eye and aqueous humour samples were taken at predetermined time points. The collected samples were then analyzed for moxifloxacin by HPLC. The aqueous humour drug concentrations as a function of time are plotted in Figure 13 and the pharmacokinetic parameters are presented in Table 4.

The C_max _achieved after instillation of 30µl of commercial eye drops or an MX 10, MX 15, and MX 20 nanoformulation was 1810 ± 59.4, 3106 ± 45.9, 2965 ± 102.1, and 2725 ± 85.2 ng/mL, respectively. The C_max_ achieved with the nanoparticle formulations was 1.5-1.71 times greater than that of the commercial eye drops.

The AUC_0-t_attained for commercial eye drops and the nanoformulations MX 10, MX 15, and MX 20 were 2640 ± 26, 8290 ± 88, 8030 ± 83, and 8410 ± 100 µg-h/mL, respectively, indicating a three-fold higher bioavailability of the drug from nanoparticle formulations when compared to that achieved with the commercial solution. The MRT for MX 10, MX 15, MX 20, and commercial eye drops was 4.06 ± 0.09, 5.1 ± 0.1, 4.7 ± 0.08, and 1.44 ± 0.04 h, respectively. 

The MRT for nanoformulations was 2.82-3.54 times higher than that of the commercial eye drops. The higher MRT of the nanoformulations suggests the influence of their enhanced ocular contact duration when compared with that of the commercial eye drops.

The drug concentration in the aqueous humour was un-detectable 3 h after instillation of the commercial eye drops, which might be due to the rapid pre-corneal drainage ensuing a shorter residence time of the drug at the ocular surface. With nanoformulations, the drug was retained up to 8 h post instillation leading to enhanced bioavailability of the drugs to the aqueous humour. The reason for this improved bioavailability may be that the PLGA nanoparticles are retained longer in the conjunctival sac (54). As the results of ANOVA, the question of which formulations were significantly different, the statistical least significance difference (LSD) test was applied. The results obtained showed that each of the calculated LSD values is smaller than the tabulated value and hence each of the nanoformulations is unique and significantly different from the other formulations and the commercial product for these pharmacokinetic parameters.

## Conclusion

Polymeric moxifloxacin hydrochloride nanoparticles based on PLGA RG 752H were prepared by a modified double emulsion-solvent evaporation method and characterized for various properties. The *in-vitro *drug release studies revealed an initial faster drug release rate in the first 6 hours followed by slower and sustained release. Each data set could be described by the Higuchi release model for data past the 6 h of burst release, identifying a diffusion release mechanism in effect in the latter phase of drug release. The stability studies indicated that the nanoformulations were stable on storage at 4 °C for 2 months. The *in-vivo *studies suggested the nanoformulations are safe and free of any irritating effects on the eye. The ocular bioavailability of nanoformulations was higher than the available commercial ophthalmic solution; this could lead to lower doses and lower costs to the patient. These nanoformulations can be adopted as an alternative to commercial eye drops because they can retain the drug at the ocular surface, reducing the dosing frequency and thus resulting in better patient compliance. However, more studies are required to evaluate the clinical efficacy of these nanoformulations.
